# The Estimation of the Number of Aborted Girls in South Korea

**DOI:** 10.1002/ajhb.70046

**Published:** 2025-04-17

**Authors:** Hyunkuk Cho

**Affiliations:** ^1^ School of Economics and Finance Yeungnam University Gyeongsangbuk‐do South Korea

**Keywords:** abortion, sex ratio at birth, sex selection, son preference

## Abstract

**Objective:**

Countries such as China and India are well known for their preference for sons. South Korea is another country with a long‐standing preference for sons, with the sex ratio at birth (SRB) reaching as high as 116.5 in some years. Given that the normal SRB ranges from 105 to 107, a higher SRB suggests that some girls were likely aborted. This study estimates the number of girls aborted in the country.

**Methods:**

Using an SRB of 106 as normal, we first calculate the expected number of girls born (NEG) based on the actual number of newborn boys (NAB). That is, NEG = (100/106) × NAB. We then compare NEG with the actual number of newborn girls.

**Results:**

In 1981, 448 655 boys were born, which would imply that 423 259 girls were expected. However, since 418 754 girls were actually born that year, 4505 girls were likely aborted. In total, approximately 340 000 girls were aborted from 1981 to 2010, accounting for 3.8% of all female births.

**Conclusion:**

We estimated the number of aborted girls based on the number of boys born. Since some boys were likely aborted, the number of aborted girls is likely higher than 340 000. Therefore, this figure should be considered as an estimate of abortions due to son preference.

## 1 Introduction

**FIGURE 1:**
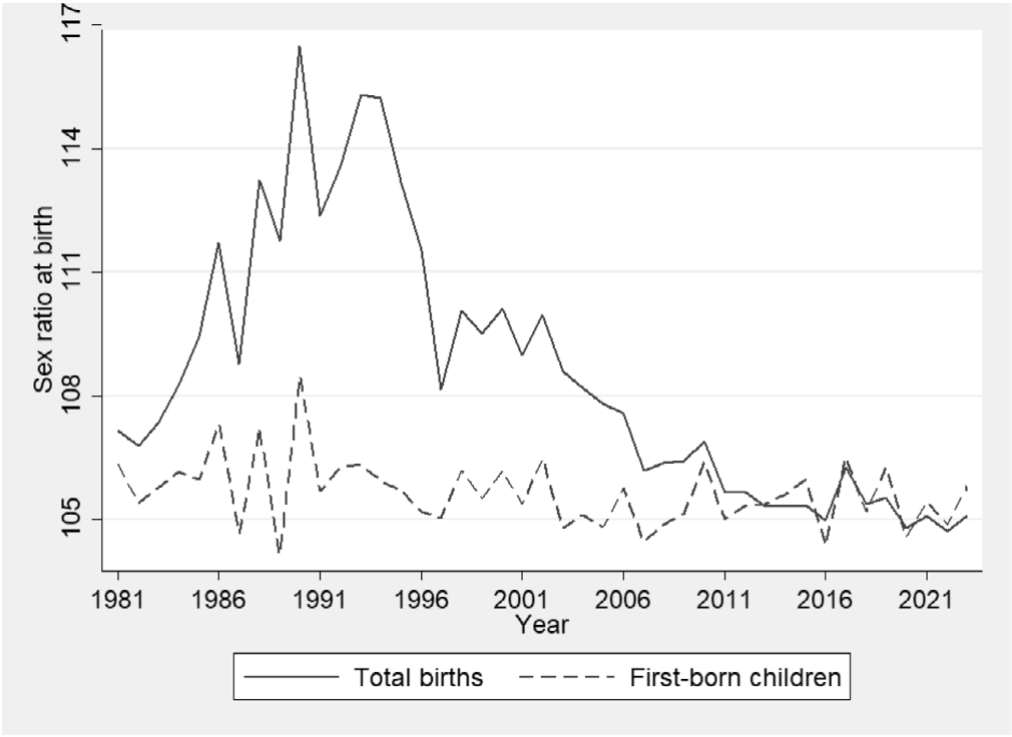
Sex ratio at birth from 1981 to 2023. The numbers in this figure were calculated by the author based on the data source provided in endnote 4.

Countries such as China and India are well‐known for their preference for sons. South Korea also has a long‐standing preference for sons, with the sex ratio at birth (SRB) reaching as high as 116.5 in some years. Figure 1 shows that the SRB increased until the mid‐1990s, then decreased to 105 in 2020. Given that the normal SRB ranges from 105 to 107, a higher SRB suggests that some girls were likely aborted during this period.

To achieve a desired number of boys, women could continue having children until they reached that number, which likely kept the SRB within a normal range.^1^ However, if fetal gender detection had been available and abortion allowed, women could have aborted unwanted female fetuses, leading to an increase in the SRB, as seen in South Korea. Although abortion was illegal in the country, it was effectively tolerated, contributing to the elevated SRB.

This study estimates the number of girls who were likely aborted due to son preference. Using an SRB of 106 as normal, we first calculate the expected number of girls born (NEG) based on the actual number of newborn boys (NAB). That is, NEG = (100/106) × NAB. For example, if 106 boys were born in a year, the normal SRB of 106 indicates that 100 girls should be born. If fewer than 100 girls were born, some girls were likely aborted. In this way, we estimate the annual number of girls aborted from 1981 to 2010, the final year with an SRB greater than 106.

Son preference is not the only factor influencing SRB; other factors could also contribute to it. According to the Trivers–Willard hypothesis, women experiencing poor conditions may be more likely to terminate male fetuses or those perceived as weaker (Trivers and Willard 1973). Studies have shown that factors like malnutrition (Anderson and Bergström 1998; Almond et al. 2010) and natural disasters such as earthquakes (Fukuda et al. 1998) can increase the number of girls being born. If a year had a lower SRB for these reasons compared to the value that would have been achieved without any girl‐increasing events, we would be underestimating the number of aborted girls. However, malnutrition and earthquakes are not serious problems in South Korea, and therefore, the bias is unlikely to be large.

Additionally, studies, such as Oster (2005), reported that women infected with the hepatitis B virus (HBV) are more likely to give birth to boys, thereby raising the SRB. However, other studies (e.g., Das Gupta 2005; Lin and Luoh 2008; Lin et al. 2012) oppose this finding. For instance, Lin and Luoh (2008) found no significant effect based on Taiwanese data, reporting that the marginal increase in the probability of having a male birth for HBV mothers relative to non‐HBV mothers is only 0.0025. In addition, Das Gupta (2005) reported that the SRB for first‐born children in China was within the normal range, and the higher sex ratio at birth was driven by higher birth orders. It is worth noting that, as shown in Figure 1, the SRB among first‐born children in South Korea does not vary significantly over time, mostly remaining in the normal range. Given that the aforementioned factors should affect all birth orders, this suggests that the SRB is primarily influenced by son preference, rather than other factors. Finally, the fact that 1990 recorded the highest SRB in Figure 1, significantly higher than the two adjacent years, is likely due to the influence of the Chinese zodiac, as 1990 was the Year of the White Horse, which was considered unfavorable for the birth of girls.

This study contributes to the literature as follows. While previous studies on the number of girls aborted focused on other Asians including Chinese and Indian groups (Sen 1990; Abrevaya 2009), few have examined South Korea.^2^ Second, this study adds to the broader literature on son preference. Most previous studies have focused on the effects of son preference or high SRBs, including on sex selection (Chen et al. 2013), girls' health (Cho 2023; Ye et al. 2024), and subjective well‐being (Li 2021). Other studies from different strands include Ibupoto et al. (2025), who examined the effect of the sex composition of children on the desire for more children, presumably sons.

## 2 Literature Review and Background Information on Son Preference in South Korea

We review studies by Sen (1990) and Abrevaya (2009). Sen (1990) reported that, due to son preference, 50 million girls were missing in China, along with another 50 million girls missing in South and West Asia, as well as North Africa. The study considered an SRB of 105 as normal for the calculation. Abrevaya (2009) reported that the number of aborted girls in the United States among Chinese third and fourth births from 1991 to 2004 is approximately 900, and the estimate for Indian third and fourth births from 1992 to 2004 is nearly 1270. The study obtained these numbers by estimating the abortion rate for girls, which is given by the formula $g=\frac{\tilde{p}-p}{\tilde{p}\left(1-p\right)}$, where $\tilde{p}$ is the boy‐birth probability, and *p* is the natural probability of a boy birth. It considered boys at birth to account for 52% as normal, which is equivalent to an SRB of 108.3 (= (0.52*n*/0.48*n*) × 100, where *n* represents the total number of births).

As table 9 of the paper indicates, when $\tilde{p}$ = 0.53 for Chinese third‐born children, the abortion rate for girls (*g*) is equal to 3.8%. Since the total number of births for this birth order is 36018, the number of girls among them is equal to 16 928 (= 36 018 × 0.47), which represents 96.2% of 17 597 girls who would have been born without any selective abortion. This implies that 669 girls (= 17 597—16 928) were aborted for this birth order. Similarly, when $\tilde{p}$ = 0.54 for Chinese forth‐born children, *g* is equal to 7.4%. Given that the total number of births for this birth order is 6802, the number of girls who would have been born is 3204. This means that 250 girls were aborted for this birth order. It is worth noting that the method is essentially the same as ours, and therefore, using this method yields the same result.^3^

South Korea has traditionally exhibited a strong preference for sons. This preference stems from the patriarchal family system established during the Choson dynasty (1392–1910), where the eldest son became the head of the family and inheritance passed through the male line, fostering a son‐centric culture (Larsen et al. 1998; Chung and Gupta 2007). However, son preference has notably declined and nearly disappeared in recent years. As described, 2010 marked the last year an SRB higher than 106 was recorded. Additionally, the percentage of married women who expressed a desire to have a son at all decreased from 18.0% in 2000 to just 5.7% in 2015 (Oh et al. 2016). Furthermore, data from the Korean General Social Survey (KGSS) shows a shift in preferences: in 2004, 36.2% of people with only one child preferred a son, while 31.9% preferred a daughter. By 2015, this had reversed, with 43.1% preferring a daughter and 35.6% preferring a son.^4^

## 3 Data and Results

The data used in this study come from the National Statistical Office, which provides information on the number of boys and girls born each year, as well as their birth orders.^5^ As shown in Table 1, in 1981, 448 655 boys and 418 754 girls were born, resulting in an SRB of 107.1. The number of newborn babies decreased thereafter. When the SRB peaked at 116.5 in 1990, 349 617 boys and 300 121 girls were born. Since the SRB remained at 106 or higher until 2010, we calculated the number of aborted girls up until that year.

**TABLE 1:** Estimated number of aborted girls.

Year (1)	Number of boys born (2)	Number of girls born (3)	Number of girls expected born (4)	Estimated number of aborted girls (5)
1981	448 655	418 754	423 259	4505
1982	438 077	410 235	413 280	3045
1983	398 194	370 961	375 655	4694
1984	350 775	324 018	330 920	6902
1985	342 506	312 983	323 119	10 136
1986	335 588	300 431	316 592	16 161
1987	325 038	298 793	306 640	7847
1988	336 203	296 889	317 173	20 284
1989	337 475	301 956	318 373	16 417
1990	349 617	300 121	329 827	29 706
1991	375 276	333 999	354 034	20 035
1992	388 573	342 105	366 578	24 473
1993	383 365	332 461	361 665	29 204
1994	386 080	335 105	364 226	29 121
1995	379 604	335 416	358 117	22 701
1996	364 433	326 793	343 805	17 012
1997	350 929	324 465	331 065	6600
1998	336 147	305 447	317 120	11 673
1999	324 409	296 259	306 046	9787
2000	335 433	304 656	316 446	11 790
2001	291 989	267 945	275 461	7516
2002	260 228	236 683	245 498	8815
2003	257 727	237 309	243 139	5830
2004	247 835	229 123	233 807	4684
2005	227 592	211 115	214 709	3594
2006	234 110	217 649	220 858	3209
2007	255 872	240 950	241 389	439
2008	240 119	225 773	226 527	754
2009	229 351	215 498	216 369	871
2010	242 901	227 270	229 152	1882

*Note:* Numbers in column (4) are calculated as (100/106) × numbers in column (2). Numbers in column (5) are the difference between columns (4) and (3).

Table 1 shows that 448 655 boys were born in 1981, which would imply that 423 259 girls (calculated as (100/106) × 448 655) were expected. However, since 418 754 girls were actually born that year, we estimate that 4505 girls (= 423 259 − 418 754) were likely aborted. Similarly, based on the 1982 data, an estimated 3045 girls were likely aborted. The year 1990 recorded the highest number of abortions, with 29 706 girls.

In total, approximately 340 000 girls were aborted from 1981 to 2010. Since 8.9 million girls were born during this period, the aborted girls account for 3.8% of all female births.

## 4 Conclusion

We estimated the number of aborted girls based on the number of boys born. Since some boys were likely aborted, the number of aborted girls is likely higher than 340 000. Therefore, this figure should be considered as an estimate of abortions due to son preference. Additionally, using a value other than 106 for the normal SRB will yield a different estimate. For instance, if we use 105 as the normal SRB, we estimate that approximately 430 000 girls were likely aborted. Using 107 as the normal SRB yields an estimate of approximately 250 000 abortions.

## Data Availability

The data that support the findings of this study are available from the corresponding author upon reasonable request.

## Endnotes

^1^
A sex‐dependent stopping rule could lead to an imbalance, resulting in either an overabundance of male or female offspring, depending on the circumstances (e.g., Grech et al. 2018).

^2^
Chao et al. (2019) includes estimates for the country. In the study, the number of girls aborted is calculated as the difference between the number of female births based on SRB without inflation and with inflation. The study uses 1982 as the starting point for inflation but excludes birth registration data before 1980 due to low data quality. Instead, it relies on survey data from the 1974 World Fertility Survey, which collected information from 5430 ever‐married women.

^3^
Based on Abrevaya (2009), the expected number of girls born can be calculated by dividing the number of girls actually born (NAG) by 1 − *g*. This equals $\frac{\mathrm{NAG}}{p/\left(1-p\right)}\times \frac{\tilde{p}}{1-\tilde{p}}=\frac{n\left(1-\tilde{p}\right)}{p/\left(1-p\right)}\times \frac{\tilde{p}}{1-\tilde{p}}=\frac{100}{p/\left(1-p\right)\times 100}\times n\tilde{p}$, where *n* is the total number of births, $n\tilde{p}$ is the number of boys born, and *p*/(1 − *p*) × 100 represents the normal SRB. The final part of the equation is the same as what is used in the present study.

^4^
The data can be downloaded from https://www.icpsr.umich.edu/icpsrweb/ICPSR/series/288.

^5^
The data can be downloaded from https://kosis.kr/statHtml/statHtml.do?orgId=101&tblId=DT_1B80A01&conn_path=I3.
